# Genetic predisposition to type 2 diabetes is associated with impaired insulin secretion but does not modify insulin resistance or secretion in response to an intervention to lower dietary saturated fat

**DOI:** 10.1007/s12263-012-0284-8

**Published:** 2012-02-21

**Authors:** Celia G. Walker, Ruth J. F. Loos, Adrian P. Mander, Susan A. Jebb, Gary S. Frost, Bruce A. Griffin, Julie A. Lovegrove, Thomas A. B. Sanders, Les J. Bluck

**Affiliations:** 1 MRC Human Nutrition Research, Elsie Widdowson Laboratory, Fulbourn Road, Cambridge, CB1 9NL UK; 2 MRC Epidemiology Unit, Institute of Medical Science, Addenbrooke’s Hospital, Cambridge, UK; 3 MRC Biostatistics Unit Hub for Trials Methodology Research, Institute of Public Health, Cambridge, UK; 4 Nutrition and Dietetic Research Group, Imperial College London, London, UK; 5 Faculty of Health and Medical Sciences, University of Surrey, Guildford, UK; 6 Hugh Sinclair Unit of Human Nutrition, Institute for Cardiovascular and Metabolic Research (IMCR), University of Reading, Reading, UK; 7 Nutritional Sciences Division, Kings College London, London, UK

**Keywords:** Dietary intervention, SNP, Insulin resistance, Insulin secretion, Saturated fat, IVGTT

## Abstract

Genome-wide association studies have identified SNPs reproducibly associated with type 2 diabetes (T2D). We examined the effect of genetic predisposition to T2D on insulin sensitivity and secretion using detailed phenotyping in overweight individuals with no diagnosis of T2D. Furthermore, we investigated whether this genetic predisposition modifies the responses in beta-cell function and insulin sensitivity to a 24-week dietary intervention. We genotyped 25 T2D-associated SNPs in 377 white participants from the RISCK study. Participants underwent an IVGTT prior to and following a dietary intervention that aimed to lower saturated fat intake by replacement with monounsaturated fat or carbohydrate. We composed a genetic predisposition score (T2D-GPS) by summing the T2D risk-increasing alleles of the 25 SNPs and tested for association with insulin secretion and sensitivity at baseline, and with the change in response to the dietary intervention. At baseline, a higher T2D-GPS was associated with lower acute insulin secretion (AIRg 4% lower/risk allele, *P* = 0.006) and lower insulin secretion for a given level of insulin sensitivity, assessed by the disposition index (DI 5% lower/risk allele, *P* = 0.002), but not with insulin sensitivity (Si). T2D-GPS did not modify changes in insulin secretion, insulin sensitivity or the disposition index in response to the dietary interventions to lower saturated fat. Participants genetically predisposed to T2D have an impaired ability to compensate for peripheral insulin resistance with insulin secretion at baseline, but this does not modify the response to a reduction in dietary saturated fat through iso-energetic replacement with carbohydrate or monounsaturated fat.

**Electronic supplementary material:**

The online version of this article (doi:10.1007/s12263-012-0284-8) contains supplementary material, which is available to authorized users.

## Introduction

Type 2 diabetes (T2D) results from a combination of insulin resistance and impaired ability of the pancreatic beta-cell to secrete sufficient insulin (Kahn 2003). Diet composition, especially the amount and type of dietary fat, is a recognised environmental risk factor for T2D, which particularly affects peripheral insulin sensitivity (Parillo and Riccardi 2004). The quality of dietary carbohydrate is also important with a higher glycaemic index (GI) diet being associated with an increased risk of T2D (Barclay et al. 2008). Genome-wide association studies (GWAS) have identified loci that show robust association with increased risk of developing T2D (Saxena et al. 2007; Sladek et al. 2007; Voight et al. 2010; Zeggini et al. 2007) and with glycaemic traits (Dupuis et al. 2009). Most of these SNPs reside in or near genes that have a presumed role in pancreatic beta-cell dysfunction (Billings and Florez 2010). These loci have mostly been identified using overt measures of impaired glucose tolerance or frank diabetes; however, the impact on insulin sensitivity and beta-cell function has only been assessed using proxy measures. More detailed phenotyping has the advantage of identifying early-stage defects that exist before impaired fasting glucose becomes apparent.

In this study, we examined the effect of a T2D genetic predisposition score (GPS) on beta-cell function and peripheral insulin sensitivity, assessed by intravenous glucose tolerance test (IVGTT) in a cohort of overweight non-diabetic participants at increased cardiometabolic risk (Jebb et al. 2010). This was in order to determine the effect of SNPs that have been most robustly associated with T2D in GWAS on early-stage defects that can precede T2D. Furthermore, we examined whether this genetic predisposition to T2D influenced changes in insulin sensitivity and beta-cell function in response to changes in dietary fat and carbohydrate intake. This was tested in participants in a 24-week dietary intervention study to lower saturated fat intake through iso-energetic replacement with low or high GI carbohydrate or monounsaturated fat (MUFA). The objective of this analysis was to assess whether the T2D-GPS can act to modify dietary associated changes in peripheral insulin sensitivity and beta-cell function.

## Methods

### Original RISCK trial study design

The RISCK trial (ISRCTN29111298) has been described in detail previously (Jebb et al. 2010). Briefly, men and women aged 30–70 years (*n* = 720) at increased cardiometabolic risk (according to a study-specific scoring system) but with no diagnosis of T2D were recruited from the general population (Jebb et al. 2010). All participants underwent a 4-week run-in period on the ‘reference’ diet (REF), designed to reflect the high saturated fat intake of a ‘Western diet’. Participants were then randomised to the REF diet, or one of four diets designed to achieve a target reduction in saturated fat intake from ~18% of energy (REF diet) to ~10% of energy, for 24 weeks; the actual mean reduction achieved was 7–8% (Jebb et al. 2010). The REF and intervention diets described in detail previously (Moore et al. 2009) were designed to be iso-energetic, but varied in the amount and type of fat and carbohydrate as follows: high saturated fat and high GI (REF); high MUFA/high GI; high MUFA/low GI; low fat/high GI; and low fat/low GI diets. Measurements taken after the run-in REF diet are referred to in this study as ‘baseline’ measurements. At baseline, and following the dietary intervention, a fasting blood sample was collected, anthropometry measured, and an IVGTT performed.

Ethical approval for the RISCK study was granted from the National Research Ethics Service and written informed consent including subsequent genetic analyses was obtained from participants.

### Study cohort

Of the 720 participants, 549 completed the study, and DNA was available for 512 participants. To reduce heterogeneity in genetic background, 412 individuals of white European ancestry, based on self-reported ethnicity, were included in the analysis. All other ethnic sub-groups were excluded from these analyses due to their limited size. Following genotyping quality control procedures (see below), 405 participants were available for analysis at baseline, and 376 completed the dietary intervention. Data from the IVGTT was available for 377 participants at baseline and for 354 of those who completed the study.

Of these participants, 48% had fasting glucose >5.6 mM (impaired fasting glucose), and no participants had fasting glucose >7 mM (T2D). The characteristics of the participants included in these analyses are presented in Table 1.

**Table 1:** Characteristics of participants who were analysed at baseline and at the end of the study

	Participants analysed at baseline^1^	Participants who completed study^2^
*n*	377	354
Age	53.2 (9.9)	53.5 (10)
Female (%)	58	57
BMI	28.8 (4.6)	28.7 (4.5)
Fasting glucose	5.5 (0.6)	5.5 (0.6)
Fasting plasma glucose >5.6 (%)	48	49

Characteristics of participants at entry into the study, who were included in the analysis of association of T2D-GPS with (1) traits at baseline and (2) the change in traits following the dietary intervention. Data are presented as mean (SD) or per cent

### IVGTT

A short IVGTT protocol was used and described in more detail elsewhere (Jebb et al. 2010). The area under the plasma insulin curve up to 19 min was computed to indicate the degree of endogenous insulin secretion in response to the glucose challenge (AIRg). Insulin sensitivity (Si) was estimated using the MINMOD Millennium programme (Version 6.02). The disposition index (DI) was calculated as the product of Si and AIRg and is a measure of the beta-cell’s ability to compensate for changes in Si (Bergman et al. 2002).

### SNP selection and genotyping

Twenty-eight SNPs were identified from GWAS to be associated with T2D risk (Dupuis et al. 2009; Saxena et al. 2007; Sladek et al. 2007; Voight et al. 2010; Zeggini et al. 2007). SNPs were only selected from GWAS with at least 1,000 individuals in the discovery stage, which after follow-up reached the threshold of genome-wide significance of *P* < 5 × 10^−8^. Where multiple SNPs resided in or near the same gene, only SNPs in low linkage disequilibrium (LD *r* ^2^ < 0.3) were selected.

Genotyping was performed by KBiosciences (Hoddesdon, Herts, UK) using a fluorescence-based competitive allele-specific PCR (KASPar) technology, and all SNPs had a call rate >95%. Individuals were excluded if genotyping was unsuccessful in >10% of SNPs (*n* = 7). Genotype distributions of all SNPs were tested for deviation from the Hardy–Weinberg Equilibrium using the log-likelihood ratio Chi-square test (1 *df*) for association. Using a cut-off of *P* < 0.001 excluded three SNPs (rs1470579-IGFBP2; rs13266634-SLC30A8; rs8042680-VPS33B) from analyses due to deviation. The remaining 25 SNPs in or near 24 genes were included in the current analyses (Table 2).

**Table 2:** T2D-SNPs that compose the GPS and the association with insulin sensitivity (Si), insulin secretion (AIRg) and disposition index (DI) at baseline

SNP	Near gene	Location/type	Risk Allele for T2D	Risk-allele Frequency	Insulin sensitivity (Si)			Insulin secretion (AIRg)			Disposition index (DI)		
					Effect of risk allele	SE	*P*	Effect of risk allele	SE	*P*	Effect of risk allele	SE	*P*
rs4607103	ADAMTS9	Intergenic	C	0.72	−0.10	0.04	0.01	0.04	0.07	0.57	−0.06	0.07	0.45
rs243021	BCL11A	Intergenic	A	0.47	−0.03	0.04	0.38	−0.02	0.06	0.75	−0.05	0.06	0.44
rs7754840	CDKAL1	Intron	C	0.30	0.06	0.04	0.15	−0.02	0.07	0.77	0.03	0.07	0.66
rs10811661	CDKN2A/B	Intergenic	T	0.84	0.01	0.05	0.80	0.05	0.08	0.50	0.07	0.08	0.41
rs1552224	CENTD2	Intergenic	A	0.86	−0.02	0.05	0.74	0.04	0.08	0.62	0.03	0.09	0.75
rs9939609	FTO	Intron	A	0.39	0.03	0.04	0.48	−0.07	0.06	0.25	−0.05	0.07	0.41
rs780094	GCKR	Intron	C	0.36	−0.01	0.04	0.76	−0.03	0.06	0.59	−0.04	0.06	0.50
rs1111875	HHEX/IDE	Intergenic	C	0.59	0.01	0.04	0.88	−0.06	0.06	0.38	−0.06	0.07	0.40
rs1531343	HMGA2	Intergenic	C	0.12	−0.06	0.06	0.31	−0.04	0.10	0.72	−0.11	0.11	0.31
rs7957197	HNF1A	Intron	T	0.16	−0.03	0.05	0.48	−0.10	0.08	0.20	−0.13	0.08	0.11
rs4430796	HNF1B(TCF2)	Intron	G	0.05	0.00	0.03	0.93	−0.07	0.06	0.22	−0.07	0.06	0.22
rs7578326	IRS1	Intergenic	A	0.66	−0.05	0.04	0.24	0.01	0.07	0.84	−0.02	0.07	0.73
rs864745	JAZF1	Intron	T	0.55	−0.08	0.04	0.02	−0.06	0.06	0.33	−0.15	0.06	0.02
rs5215	KCNJ11	Missense	C	0.35	0.03	0.04	0.38	0.04	0.06	0.55	0.06	0.06	0.33
rs231362	KCNQ1	Intron	G	0.59	0.05	0.03	0.13	−0.05	0.05	0.34	−0.00	0.05	0.96
rs163184	KCNQ1	Intron	G	0.46	−0.03	0.04	0.35	−0.04	0.06	0.53	−0.07	0.06	0.27
rs1387153	MTNR1B	Intergenic	T	0.29	0.02	0.04	0.60	−0.09	0.07	0.19	−0.06	0.07	0.37
rs10923931	NOTCH2	Intron	T	0.14	−0.02	0.06	0.76	−0.14	0.10	0.17	−0.17	0.10	0.11
rs1801282	PPARG	Missense	C	0.91	−0.03	0.06	0.58	0.20	0.10	0.05	0.18	0.11	0.09
rs7901695	TCF7L2	Intron	C	0.32	0.04	0.04	0.36	−0.26	0.06	0.00005	−0.23	0.07	0.001
rs7578597	THADA	Missense	T	0.88	−0.04	0.05	0.48	0.11	0.09	0.26	0.07	0.10	0.48
rs7961581	TSPAN8/LGR5	Intergenic	C	0.25	0.03	0.04	0.43	0.02	0.07	0.82	0.04	0.08	0.64
rs1801214	WFS1	Missense	T	0.59	−0.06	0.04	0.12	0.03	0.06	0.63	−0.03	0.06	0.61
rs4457053	ZBED3	Intergenic	G	0.31	0.04	0.04	0.21	−0.07	0.06	0.26	−0.03	0.06	0.66
rs11634397	ZFAND6	Intergenic	G	0.63	−0.01	0.04	0.83	−0.00	0.07	0.94	−0.02	0.07	0.79

Data are the coefficient of associations presented as per allele effect size, standard error and *P* derived from linear regression models. The linear regression models were of SNP against Si, AIRg or DI at baseline adjusted for age, gender, BMI and centre. Data for Si, AIRg and DI were log(*n*)-transformed for analysis and are presented in this form

### Genetic predisposition score

We defined the risk allele of a SNP as the allele associated with increased risk of developing T2D or with raised fasting plasma glucose concentration in previous GWAS (Dupuis et al. 2009; Saxena et al. 2007; Sladek et al. 2007; Voight et al. 2010; Zeggini et al. 2007). An individual’s genotype was coded as 0, 1 or 2 depending on the number of the risk alleles an individual carried for that particular SNP. For each individual, a GPS was calculated by adding the number of risk alleles of the 25 SNPs (Table 2). As there is currently no evidence for interaction between SNPs, a simple addition of the associated risk alleles for each trait has been commonly adopted (Hamrefors et al. 2010; Li et al. 2010; Talmud et al. 2009). For participants with missing genotypes (<10%), the average count of risk alleles for the respective SNP was substituted for the missing genotype for the purpose of calculating the GPS. The GPS was normally distributed. The SNPs selected for analysis are presented in Table 2 showing the risk-allele frequency.

### Statistical analysis

Distributions of traits were tested for normality; and because of right skewness Si, AIRg and DI were natural log-transformed for analyses and presented in figures as the geometric mean and 95% confidence intervals. For interpretation of the effect of T2D-GPS on traits, the coefficient of association from the linear regression analysis of log(*n*)-transformed traits equated to the percentage change per risk allele.

Due to insufficient power to examine associations of individual SNPs, we focussed our study on the GPS, which provides more power. Linear regression analysis was used to test for associations between GPS (a continuous variable according to the number of risk alleles) and traits at baseline, assuming an additive effect of each additional risk allele, while adjusting for age, gender, centre and BMI. We additionally tested for curvature in the model by inclusion of GPS-squared term in the model.

Next, we tested for the effect of GPS to modify the change in Si, AIRg and DI following 24 weeks of dietary intervention by an interaction between T2D-GPS and dietary intervention group. This was used in a linear regression model of the association between the GPS and change in Si, AIRg and DI following intervention, adjusted for baseline values, age, gender, centre, baseline BMI, diet and change in weight. The four intervention diets were compared to the REF diet group.

Associations between the individual SNPs and traits at baseline, and the interaction effects of SNPs and dietary intervention group on change in trait in response to the dietary intervention, were tested with linear regression in the same way GPS were tested, adjusting for the same covariates. This exploratory analysis was conducted despite the assumption that we would have low power to detect small effects of individual SNPs to illustrate the contribution to the effects of GPS.

Statistical analysis was conducted using Stata 11 (StataCorp, TX, USA). A Bonferroni correction was applied to the six tests between T2D-GPS and the three traits at baseline, and to change in response to the intervention (*P* = 0.008). We did not correct the associations of individual SNPs as we decided to only report the summary statistics for future research, rather than to interpret them on their own due to the limited statistical power to detect small individual effects when corrected for false positive chance.

## Results

**Fig. 1:**
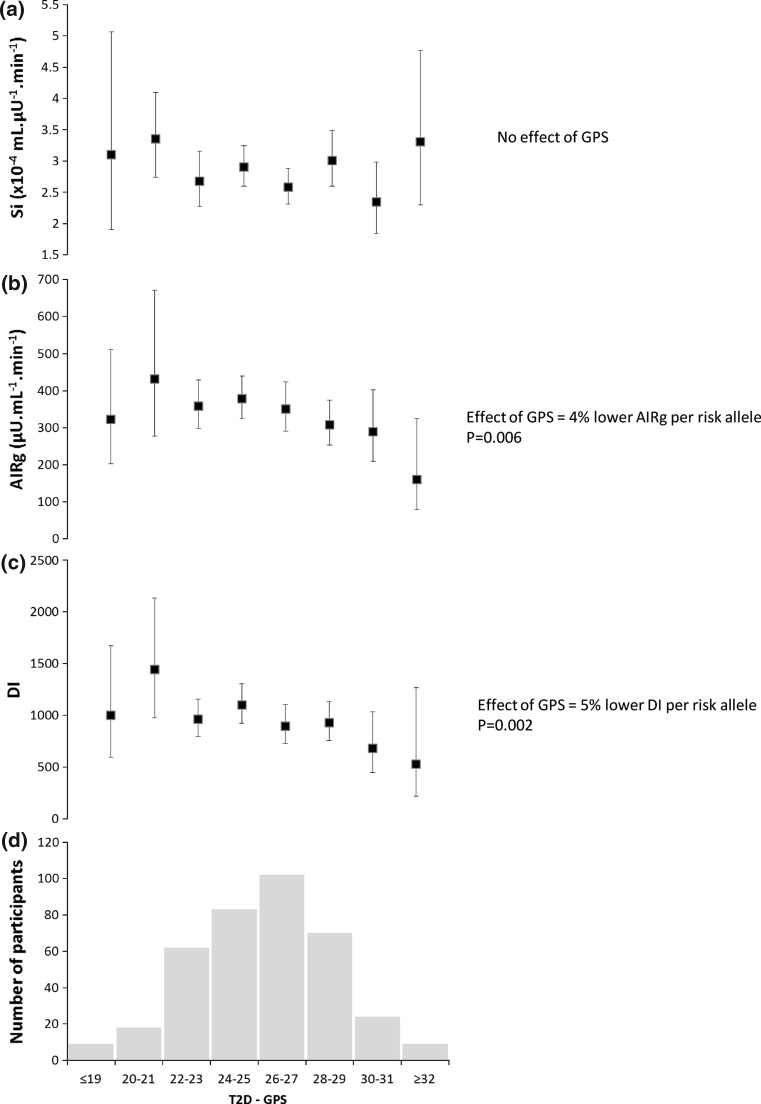
Effect of type 2 diabetes genetic predisposition score (T2D-GPS) on insulin sensitivity (Si), acute insulin secretion (AIRg) and disposition index (DI) at baseline. The participants were stratified by T2D-GPS, and the number of participants in each GPS stratum is shown in panel** d**. The effect of T2D-GPS on (**a**) Insulin sensitivity index (Si) (**b**) Acute insulin secretion (AIRg) and (**c**) Disposition index (DI) is presented as geometric mean and 95% CI. Data were log(*n*)-transformed for analysis, and the per allele effect was determined by linear regression analysis with age, gender, BMI and centre as confounding variables. The effect is presented as the beta-coefficient from the linear regression of the log(*n*)-transformed trait (which equated to percentage difference) and the *P* value. These measures were conducted after a 1-month period on a ‘reference’ high SFA diet prior to the dietary intervention to lower SFA intake

At baseline, we observed no association of T2D-GPS with peripheral insulin sensitivity (Si) (Fig. 1a). However, the T2D-GPS was associated with lower acute insulin secretion (4% per risk allele, *P* = 0.006, Fig. 1b) and with a lower disposition index (5% per risk allele, *P* = 0.002, Fig. 1c).

Of all the SNPs tested, only the TCF7L2 SNP rs7901695 (Table 2) showed a convincing association with both AIRg (13% lower per risk allele, *P* = 0.00005) and DI (11% lower per risk allele, *P* = 0.001), but not with Si. To examine whether the associations observed for the GPS with AIRg and DI were driven by the effect of the TCF7L2 SNP, we excluded this SNP from the GPS and tested for association again. The associations remained for AIRg (3% lower per risk allele, *P* < 0.05) and DI (4% lower per risk allele, *P* = 0.02), but the effect was diminished, and they were no longer significant when accounting for multiple testing.

In response to the 24 week dietary interventions to lower saturated fat intake, there were no effects of T2D-GPS to modify the change in Si, AIRg or DI, accounting for baseline values, in response to any of the dietary interventions (Table 3).

**Table 3:** The association of T2D-GPS with change in insulin sensitivity (Si), insulin secretion (AIRg) and disposition index (DI) in response to the dietary interventions to lower saturated fat intake

	Per allele effect of T2D-GPS on change in trait on the reference diet			Per allele effect of T2D-GPS on change in trait on the High MUFA/high GI diet			Per allele effect of T2D-GPS on change in trait on the High MUFA/low GI diet			Per allele effect of T2D-GPS on change in trait on the Low fat/high GI diet			Per allele effect of T2D-GPS on change in trait on the Low fat/low GI diet			Global test of diet
	Effect	SE	*P*	Effect	SE	*P*	Effect	SE	*P*	Effect	SE	*P*	Effect	SE	*P*	*P*
Change in insulin sensitivity (Si)	0.00	0.02	0.93	0.00	0.02	0.92	−0.03	0.02	0.11	−0.02	0.01	0.16	0.02	0.02	0.19	0.24
Change in insulin secretion (AIRg)	−0.02	0.03	0.58	−0.02	0.02	0.44	−0.02	0.02	0.31	−0.01	0.02	0.46	−0.01	0.02	0.46	0.99
Change in disposition index (DI)	−0.02	0.03	0.63	−0.01	0.02	0.58	−0.04	0.02	0.05	−0.03	0.02	0.14	0.00	0.02	0.87	0.61

Data are presented as per allele effect size of T2D-GPS on the change in trait by each dietary intervention group, derived from the coefficient, standard error and *P* value of the T2D-GPS (reference) and T2D-GPS + interaction terms for each diet group from linear regression models. The models were of T2D-GPS against change in Si, AIRg or DI, adjusted for baseline measure, age, gender, BMI, centre, weight change and diet group and T2D-GPS × diet group. Data for Si, AIRg and DI were log(*n*)-transformed for analysis and are presented in this form

Of all the SNPs tested, no convincing effect was observed on the change in Si, AIRg or DI in response to any of the dietary interventions (Supplementary Table 1).

## Discussion

This study used detailed phenotyping from the IVGTT to characterise the effects of genetic predisposition to T2D on insulin secretion and sensitivity in non-diabetic overweight participants at increased cardiometabolic risk. Our results show that at baseline, a genetic predisposition to T2D was associated with an impairment of beta-cell function, but not with insulin sensitivity in participants on a run-in diet high in saturated fat, designed to reflect a ‘Western diet’. However, genetic predisposition to T2D did not modify changes in insulin sensitivity or beta-cell function in response to a dietary intervention to lower saturated fat intake by isoenergetic replacement with MUFA or carbohydrate.

Previous GWAS have identified genetic loci associated with T2D, the majority of which have a presumed role in beta-cell dysfunction (Billings and Florez 2010). Our findings are supportive of a mechanism contributing to impaired insulin secretion but not insulin sensitivity. The DI is a more informative measure of beta-cell function than acute insulin secretion; by taking into account the insulin sensitivity it is a measure of the ability of the beta-cell to compensate for the degree of insulin sensitivity (Bergman et al. 2002). This measure can also be more sensitive as differences in DI can be detected before changes in acute insulin secretion are apparent (Bergman et al. 2002). At baseline, genetic predisposition to T2D was associated with a lower acute insulin response, but also with a lower DI, which confirmed an impaired acute insulin response for the level of insulin resistance.

The dietary modifications in the RISCK study were designed to be achievable through simple dietary modification and implementable on a population scale, but produced no overall significant effect on insulin sensitivity (Jebb et al. 2010). Here we demonstrate that genetic predisposition to T2D did not moderate any effects of the dietary intervention on changes in insulin secretion or sensitivity. An effect of genetic predisposition on changes in these measures may have been evident with a more extreme intervention, particularly one that also achieved weight loss. For example, in response to a lifestyle intervention to decrease caloric intake and increase energy expenditure (TULIP, *n* = 1576), carriers of the rs7903146 (TCF7L2) risk allele, who had impaired glucose tolerance prior to the intervention, showed an increase in post-glucose-load insulin secretion (adjusted for change in BMI), which was not shown in non-carriers (Heni et al. 2010). The same locus had also previously been associated with increased risk of progression to T2D, which was lessened by metformin therapy in the diabetes prevention programme (DPP), a larger multi-ethnic cohort at high risk of developing T2D (*n* = 3548) (Florez et al. 2006). Several other SNPs were also identified to have nominal interactions with metformin on incidence of T2D (*n* = 2994) (Jablonski et al. 2010) or in treatment response (*n* = 3920) (Zhou et al. 2011). However, also in participants of the DPP (*n* = 2843), insulin sensitivity indices were studied using a genetic risk score (analogous to our GPS and with 23 of 34 SNPs covered in our study). Similar to the current study, they found a trend for a lower estimated insulin secretion (insulinogenic index) and oral DI (from a 2 h post-oral glucose load value) at baseline, but there was also no overt effect of GPS on change in insulin sensitivity or secretion indices following 1 year of intensive lifestyle modification or metformin treatment (Hivert et al. 2011). It appears that SNPs that are more strongly associated with a given trait in cross-sectional data may not be the most important SNPs in terms of change in trait, especially when improvements are seen in the trait that underlies the association.

A meta-analysis conducted by MAGIC (Meta-Analysis of Glucose- and Insulin-related traits Consortium) identified SNPs associated with increased glycaemia and insulin resistance in non-diabetic participants, using surrogate measures of beta-cell function (HOMA-B) and insulin sensitivity (HOMA-IR) in >35,000 participants (Dupuis et al. 2009). MAGIC identified more than twelve robust associations with fasting plasma glucose and beta-cell function, but only two with HOMA-IR or fasting insulin as a measure of insulin sensitivity. However, the studies included in this meta-analysis used proxy measures of insulin sensitivity and secretion, whilst in the current study, we used detailed phenotyping from the IVGTT to fully characterise insulin secretion and sensitivity by genetic predisposition to T2D, albeit in a small number of participants. Furthermore, examining these parameters before and in response to a dietary intervention showed that the effect of this genetic predisposition did not change the ability to respond to environmental changes. Only a small number of studies have examined the cumulative effect of loci most strongly associated with risk of T2D in response to an intervention or over time. The effects of a combined GPS in the DPP are discussed above (Hivert et al. 2011). An increased T2D risk score, composed of four alleles, has been associated with an accelerated age-related decline in beta-cell function in a longitudinal study (Haupt et al. 2009). In this case, a combined GPS from SNPs associated with 2 h post-load glucose was shown to contribute to a steeper age-related decline in glucose tolerance (Jensen et al. 2011).

Despite the small sample size and potential insufficient statistical power to examine effects of individual SNPs, in an exploratory analysis, we found significant associations of the TCF7L2 SNP with lower acute insulin secretion and DI, but no association between this SNP and the change in insulin secretion or DI in response to the intervention. The sensitivity analysis, in which we excluded the TCF7L2 SNP from the GPS, indicated that the accumulation of risk alleles, other than TCF7L2, still contributed to the impairment of beta-cell function, although the effect was not as strong. In participants from the DPP (*n* = 3548), the T2D risk-conferring allele of TCF7L2, which was associated with proxy measures of insulin secretion, but not sensitivity at baseline, also found no association with change in these measures in response to metformin or lifestyle interventions (Florez et al. 2006).

In conclusion, using detailed phenotyping from IVGTT, we demonstrated that genetic predisposition to T2D is associated with impaired beta-cell function in non-diabetic, overweight participants on a high saturated fat diet, which reflects an average ‘Western diet’. This genetic predisposition did not moderate effects of a reduction in dietary saturated fat by replacement with MUFA or carbohydrates on changes in insulin sensitivity or beta-cell function.

## Electronic supplementary material

Below is the link to the electronic supplementary material.

Supplementary material 1 (PDF 403 kb)
