# Supplementary material for: Genetic predisposition to type 2 diabetes is associated with impaired insulin secretion but does not modify insulin resistance or secretion in response to an intervention to lower dietary saturated fat
Source: Genes Nutr. 2012 Feb 21;7(4):529–36. doi: 10.1007/s12263-012-0284-8 (PMC3448035; doi:10.1007/s12263-012-0284-8)
Supplement: Supplementary file 1 — Supplementary material 1 (PDF 403 kb) [file 12263_2012_284_MOESM1_ESM.pdf]

Supplementary Table 1. The association of each SNP with change in insulin sensitivity (Si), insulin secretion (AIRg) and disposition index (DI) in response to the dietary interventions to lower saturated fat intake

		Per allele effect of SNP on change in trait on the reference diet			Per allele effect of SNP on change in trait on the High MUFA/High GI diet			Per allele effect of SNP on change in trait on the High MUFA/low GI diet			Per allele effect of SNP on change in trait on the Low fat/high GI diet			Per allele effect of SNP on change in trait on the Low fat/low GI diet			Global test of diet
		Effect	SE	P	Effect	SE	P	Effect	SE	P	Effect	SE	P	Effect	SE	P	
Insulin sensitivity																	
rs4607103	ADAMTS9	-0.11	0.08	0.23	-0.05	0.08	0.59	0.11	0.09	0.22	0.01	0.07	0.84	0.14	0.08	0.09	0.22
rs243021	BCL11A	0.03	0.08	0.75	-0.03	0.06	0.70	-0.05	0.08	0.54	0.00	0.07	0.97	0.00	0.07	0.95	0.97
rs7754840	CDKAL1	0.05	0.10	0.63	-0.04	0.08	0.64	0.10	0.07	0.17	0.07	0.08	0.37	0.03	0.07	0.69	0.78
rs10811661	CDKN2A/B	-0.15	0.11	0.17	-0.02	0.09	0.82	0.08	0.09	0.42	0.00	0.08	0.97	0.11	0.09	0.24	0.41
rs1552224	CENTD2	0.08	0.14	0.57	0.00	0.09	0.99	0.05	0.11	0.64	0.09	0.10	0.40	0.02	0.08	0.82	0.96
rs9939609	FTO	0.04	0.08	0.61	-0.05	0.07	0.51	-0.08	0.08	0.29	-0.11	0.07	0.10	-0.01	0.08	0.91	0.62
rs780094	GCKR	0.06	0.09	0.46	-0.05	0.07	0.45	-0.06	0.07	0.36	-0.06	0.07	0.40	0.06	0.07	0.37	0.51
rs1111875	HHEX/IDE	0.03	0.08	0.75	0.03	0.07	0.71	-0.15	0.08	0.06	-0.06	0.07	0.37	0.09	0.08	0.23	0.21
rs1531343	HMGA2	-0.01	0.14	0.94	0.25	0.14	0.08	-0.04	0.10	0.66	0.02	0.11	0.87	0.17	0.11	0.13	0.38
rs7957197	HNF1A	0.01	0.17	0.95	-0.04	0.08	0.63	-0.12	0.09	0.17	0.02	0.10	0.82	0.04	0.09	0.66	0.72
rs4430796	HNF1B(TCF2)	0.04	0.08	0.65	-0.04	0.07	0.58	-0.10	0.06	0.14	-0.02	0.06	0.69	0.05	0.07	0.44	0.55
rs7578326	IRS1	0.07	0.08	0.40	-0.06	0.07	0.41	-0.07	0.08	0.37	-0.05	0.07	0.51	0.05	0.09	0.59	0.63
rs864745	JAZF1	0.00	0.08	0.96	-0.07	0.08	0.37	-0.05	0.07	0.47	0.08	0.07	0.26	0.00	0.07	0.98	0.62
rs5215	KCNJ11	-0.05	0.08	0.49	-0.06	0.08	0.48	0.08	0.07	0.25	-0.17	0.07	0.02	-0.11	0.07	0.12	0.14
rs231362	KCNQ1	-0.19	0.08	0.03	0.03	0.06	0.67	0.01	0.06	0.86	0.01	0.07	0.93	-0.07	0.07	0.28	0.27
rs163184	KCNQ1	-0.01	0.08	0.89	0.08	0.07	0.28	-0.01	0.08	0.90	-0.11	0.07	0.10	-0.01	0.08	0.86	0.45
rs1387153	MTNR1B	0.03	0.10	0.76	0.04	0.07	0.59	0.08	0.08	0.35	0.05	0.08	0.50	0.05	0.08	0.55	0.99
rs10923931	NOTCH	0.21	0.13	0.11	-0.02	0.10	0.87	-0.10	0.12	0.41	0.02	0.11	0.89	0.03	0.12	0.79	0.54

rs1801282	PPARG	-0.21	0.16	0.18	0.02	0.10	0.84	-0.16	0.13	0.21	-0.07	0.10	0.49	-0.13	0.13	0.31	0.70
rs7901695	TCF7L2	0.02	0.09	0.85	-0.03	0.07	0.71	-0.13	0.08	0.12	-0.04	0.07	0.54	0.08	0.08	0.28	0.43
rs7578597	THADA	-0.01	0.15	0.93	0.17	0.09	0.06	0.07	0.10	0.50	-0.17	0.10	0.09	0.11	0.13	0.41	0.15
rs7961581	TSPAN8/LGR5	0.03	0.10	0.74	-0.04	0.09	0.64	0.07	0.08	0.37	-0.03	0.09	0.70	-0.08	0.08	0.31	0.69
rs1801214	WFS1	0.06	0.07	0.40	-0.02	0.07	0.80	-0.16	0.08	0.04	0.05	0.07	0.46	0.10	0.07	0.16	0.13
rs4457053	ZBED3	-0.08	0.08	0.35	0.04	0.07	0.59	-0.03	0.07	0.69	0.02	0.07	0.79	0.07	0.07	0.32	0.68
rs11634397	ZFAND6	0.00	0.09	0.98	0.11	0.07	0.14	0.06	0.08	0.48	-0.04	0.07	0.58	-0.09	0.07	0.18	0.30
Insulin secretion																	
rs4607103	ADAMTS9	-0.07	0.10	0.50	0.04	0.10	0.66	-0.08	0.10	0.42	0.02	0.08	0.77	-0.01	0.09	0.88	0.86
rs243021	BCL11A	-0.03	0.09	0.76	-0.05	0.07	0.50	0.05	0.08	0.53	0.08	0.08	0.30	0.05	0.08	0.52	0.71
rs7754840	CDKAL1	-0.01	0.11	0.94	-0.05	0.09	0.60	-0.05	0.08	0.56	-0.14	0.08	0.11	-0.12	0.08	0.16	0.85
rs10811661	CDKN2A/B	-0.13	0.12	0.26	0.15	0.10	0.14	-0.32	0.10	0.00	-0.06	0.09	0.53	0.00	0.10	0.99	0.02
rs1552224	CENTD2	-0.23	0.15	0.13	-0.04	0.10	0.71	0.02	0.13	0.88	-0.08	0.12	0.49	-0.18	0.10	0.06	0.60
rs9939609	FTO	0.04	0.09	0.69	0.17	0.08	0.05	0.01	0.09	0.91	-0.07	0.07	0.40	0.03	0.09	0.77	0.37
rs780094	GCKR	0.11	0.10	0.28	0.05	0.08	0.49	-0.01	0.08	0.89	0.07	0.08	0.39	0.06	0.08	0.48	0.90
rs1111875	HHEX/IDE	-0.05	0.09	0.61	0.14	0.08	0.07	0.08	0.09	0.34	0.05	0.08	0.54	0.06	0.09	0.46	0.64
rs1531343	HMGA2	-0.12	0.16	0.45	0.06	0.16	0.73	-0.16	0.11	0.16	-0.04	0.13	0.76	0.23	0.13	0.07	0.20
rs7957197	HNF1A	-0.15	0.13	0.28	-0.09	0.10	0.34	0.11	0.10	0.26	0.04	0.11	0.72	-0.01	0.10	0.89	0.50
rs4430796	HNF1B(TCF2)	-0.09	0.09	0.35	-0.01	0.08	0.87	0.13	0.07	0.08	-0.06	0.07	0.40	0.08	0.08	0.32	0.24
rs7578326	IRS1	0.24	0.09	0.01	-0.07	0.08	0.40	0.01	0.09	0.92	-0.01	0.08	0.87	0.12	0.11	0.28	0.11
rs864745	JAZF1	-0.13	0.09	0.17	-0.03	0.09	0.73	0.01	0.08	0.88	-0.11	0.08	0.13	-0.02	0.08	0.85	0.68
rs5215	KCNJ11	0.09	0.09	0.27	-0.10	0.09	0.26	-0.06	0.08	0.43	0.00	0.08	0.99	0.02	0.08	0.81	0.55
rs231362	KCNQ1	0.08	0.09	0.36	0.10	0.07	0.12	-0.02	0.07	0.77	0.00	0.08	0.98	-0.03	0.07	0.62	0.54
rs163184	KCNQ1	-0.16	0.09	0.07	-0.12	0.08	0.12	0.03	0.09	0.73	0.03	0.08	0.70	0.01	0.09	0.92	0.32
rs1387153	MTNR1B	-0.01	0.11	0.90	0.05	0.08	0.57	-0.18	0.09	0.05	-0.13	0.09	0.12	-0.20	0.09	0.02	0.20
rs10923931	NOTCH	0.07	0.14	0.63	-0.11	0.12	0.34	0.00	0.14	0.98	-0.01	0.13	0.94	0.03	0.13	0.84	0.88
rs1801282	PPARG	-0.01	0.18	0.97	0.10	0.12	0.39	0.09	0.15	0.55	0.06	0.12	0.59	-0.11	0.15	0.48	0.83
rs7901695	TCF7L2	-0.01	0.11	0.90	-0.09	0.08	0.26	-0.06	0.09	0.54	0.03	0.08	0.76	-0.06	0.09	0.50	0.88

rs7578597	THADA	0.23	0.17	0.18	-0.16	0.10	0.11	-0.26	0.11	0.03	-0.19	0.11	0.10	0.19	0.15	0.20	0.03
rs7961581	TSPAN8/LGR5	-0.08	0.12	0.49	0.01	0.10	0.95	-0.08	0.08	0.32	-0.05	0.10	0.65	0.00	0.09	0.99	0.93
rs1801214	WFS1	-0.05	0.08	0.52	-0.02	0.08	0.79	0.12	0.09	0.19	0.03	0.08	0.69	0.01	0.08	0.88	0.69
rs4457053	ZBED3	0.09	0.09	0.33	-0.13	0.07	0.10	-0.05	0.08	0.56	0.01	0.08	0.87	-0.12	0.09	0.17	0.34
rs11634397	ZFAND6	0.02	0.09	0.82	-0.08	0.08	0.35	-0.03	0.09	0.73	0.05	0.08	0.55	-0.16	0.08	0.05	0.42
Disposition index																	
rs4607103	ADAMTS9	-0.18	0.12	0.12	-0.03	0.11	0.79	0.01	0.12	0.92	0.04	0.10	0.70	0.14	0.11	0.20	0.37
rs243021	BCL11A	0.00	0.11	0.99	-0.08	0.08	0.34	0.00	0.10	0.98	0.11	0.09	0.24	0.05	0.09	0.57	0.64
rs7754840	CDKAL1	0.03	0.13	0.84	-0.07	0.10	0.53	0.07	0.10	0.45	-0.05	0.10	0.61	-0.09	0.10	0.34	0.76
rs10811661	CDKN2A/B	-0.28	0.14	0.04	0.12	0.12	0.32	-0.25	0.12	0.04	-0.03	0.11	0.80	0.10	0.12	0.37	0.06
rs1552224	CENTD2	-0.16	0.18	0.38	-0.06	0.11	0.62	0.04	0.15	0.80	-0.05	0.14	0.71	-0.14	0.11	0.22	0.89
rs9939609	FTO	0.08	0.11	0.44	0.14	0.10	0.15	-0.04	0.10	0.66	-0.14	0.09	0.12	0.01	0.10	0.95	0.51
rs780094	GCKR	0.17	0.12	0.14	-0.01	0.09	0.95	-0.05	0.09	0.57	0.05	0.09	0.59	0.12	0.09	0.19	0.49
rs1111875	HHEX/IDE	-0.02	0.10	0.89	0.17	0.09	0.06	-0.06	0.10	0.59	0.04	0.09	0.68	0.14	0.10	0.16	0.41
rs1531343	HMGA2	-0.12	0.18	0.49	0.32	0.18	0.08	-0.18	0.13	0.17	0.00	0.15	0.99	0.41	0.14	0.005	0.02
rs7957197	HNF1A	-0.14	0.16	0.36	-0.17	0.11	0.14	-0.02	0.12	0.88	0.08	0.13	0.53	0.02	0.12	0.88	0.59
rs4430796	HNF1B(TCF2)	-0.05	0.11	0.67	-0.06	0.09	0.50	0.05	0.09	0.57	-0.07	0.08	0.43	0.13	0.09	0.16	0.48
rs7578326	IRS1	0.31	0.11	0.004	-0.14	0.09	0.13	-0.05	0.10	0.65	-0.07	0.09	0.43	0.19	0.13	0.15	0.01
rs864745	JAZF1	-0.13	0.11	0.24	-0.07	0.11	0.49	-0.04	0.09	0.63	-0.06	0.09	0.48	-0.03	0.10	0.76	0.97
rs5215	KCNJ11	0.05	0.10	0.62	-0.12	0.11	0.24	0.04	0.09	0.63	-0.18	0.10	0.06	-0.11	0.09	0.24	0.30
rs231362	KCNQ1	-0.11	0.10	0.27	0.13	0.08	0.10	-0.04	0.08	0.60	-0.03	0.09	0.77	-0.12	0.08	0.12	0.18
rs163184	KCNQ1	-0.18	0.10	0.08	-0.05	0.09	0.57	0.02	0.11	0.85	-0.04	0.09	0.64	-0.02	0.10	0.84	0.72
rs1387153	MTNR1B	0.01	0.12	0.91	0.06	0.10	0.50	-0.09	0.11	0.42	-0.09	0.10	0.35	-0.16	0.10	0.12	0.54
rs10923931	NOTCH	0.28	0.17	0.09	-0.13	0.13	0.35	-0.09	0.16	0.56	0.03	0.15	0.85	0.08	0.15	0.61	0.38
rs1801282	PPARG	-0.21	0.21	0.32	0.10	0.14	0.47	-0.09	0.17	0.60	-0.09	0.13	0.49	-0.22	0.18	0.21	0.60
rs7901695	TCF7L2	0.00	0.12	0.98	-0.11	0.09	0.22	-0.21	0.11	0.05	-0.01	0.09	0.88	0.00	0.10	1.00	0.54
rs7578597	THADA	0.23	0.20	0.24	-0.01	0.12	0.96	-0.13	0.13	0.31	-0.32	0.13	0.02	0.28	0.17	0.10	0.04
rs7961581	TSPAN8/LGR5	-0.04	0.14	0.76	-0.13	0.12	0.91	-0.03	0.10	0.79	-0.07	0.12	0.59	-0.08	0.10	0.42	0.99

rs1801214	WFS1	0.01	0.10	0.91	-0.01	0.09	0.91	-0.04	0.11	0.73	0.04	0.10	0.64	0.09	0.09	0.31	0.89
rs4457053	ZBED3	0.02	0.11	0.85	-0.07	0.09	0.43	-0.06	0.10	0.54	0.00	0.09	0.97	-0.06	0.10	0.55	0.95
rs11634397	ZFAND6	0.02	0.11	0.85	0.09	0.10	0.38	-0.01	0.11	0.91	0.02	0.09	0.83	-0.24	0.10	0.01	0.15

Data are presented as per allele effect size of individual SNPs on the effect of dietary saturated fat reduction by each diet group, derived from the coefficient, standard error and P-value of the SNP (reference) and SNP + interaction term for each diet from linear regression models. The models were of each SNP against change in Si, AIRg or DI, adjusted for baseline measure, age, gender, BMI, centre, weight change and diet group and SNP x diet group. Data for Si, AIRg and DI were log-transformed for analysis and are presented in this form.
